# Patterns of biopsy-proven renal disease in people living with HIV: 10 years experience in Sydney, Australia

**DOI:** 10.1186/s12882-022-02695-w

**Published:** 2022-04-18

**Authors:** Dane Turner, Doug Drak, David Gracey, Lyndal Anderson

**Affiliations:** 1 Department of Nephrology, Royal Prince Alfred Hospital, 50 Misseden Road Camperdown, Sydney, NSW Australia; 2 Faculty of Medicine, University of New South Wales, Sydney, NSW Australia; 3 Faculty of Medicine and Health, University of Sydney, Sydney, NSW Australia; 4 Department of Pathology, Royal Prince Alfred Hospital, Sydney, NSW Australia

**Keywords:** Australia, Biopsy, HIV, Kidney Diseases, Nephritis

## Abstract

**Background:**

Acute and chronic kidney diseases are important comorbidities in People Living With HIV (PLWH). Biopsy is often pursued in this cohort with ongoing renal impairment without a clear aetiology, in order to establish the diagnosis and to guide management. Despite the importance of renal disease in PLWH, there is a paucity of biopsy data—especially in the Australian setting. Consequently, who and when to biopsy is mainly based on clinical experience. The aims of this study were to describe biopsy-proven renal disease in PLWH at our institution and to assess for correlation between any demographic or laboratory characteristics with histological diagnosis.

**Methods:**

A retrospective review of all PLWH who underwent renal biopsy between January 2010 and December 2020 at Royal Prince Alfred Hospital, Sydney, Australia was performed. All PLWH over 18 years, who were not transplant recipients were included. Demographic, laboratory and biopsy data was extracted from the electronic medical records. Basic descriptive statistics were performed, and correlation was assessed using chi square and Kendall’s coefficient of rank test.

**Results:**

19 renal biopsies were included in the study. The majority of PLWH were Australian born (53%), male (84%) and had a mean age of 48 years (SD 13). Comorbid hypertension and diabetes were present in 74% and 21% of people respectively. The mean serum creatinine was 132 µmol/L (SD 55) and the mean estimated glomerular filtration rate (eGFR) was 61 ml/min/1.73m^2^ (SD 24). The most common histological diagnosis was tubulointerstial nephritis in 5 people (24%). Hypertensive glomerulosclerosis and IgA nephropathy were present in 4 (19%) and 3 (14%) people respectively. There were no cases of HIV-associated nephropathy. There was no significant correlation between any cohort characteristics and diagnoses.

**Conclusions:**

This study represents the first description of biopsy-proven kidney disease in the HIV-infected population of Australia. Our results support the use of renal biopsy in PLWH with ongoing renal impairment for accurate diagnosis and to guide further management. Although a small sample size, our study is larger than other published international biopsy studies.

## Background

In 2017, it was estimated that there were over 27,000 People Living With Human Immunodeficiency Virus (PLWH) in Australia [1]. The advent of highly active Anti-Retroviral Therapy (ART) has improved survival for these people and the clinical focus has now shifted to the management of comorbid conditions and adverse side effects from long-term ART use. Renal disease, although being one of the first comorbid conditions to gain significant attention, remains an important problem—with a large spectrum of acute and chronic kidney disease presentations in this population [2]. Despite the significance of kidney disease in Australian PLWH, there is a paucity of studies that investigate biopsy-proven disease in this cohort. Furthermore, previously reported international studies may not be representative of the Australian HIV population.

In addition to the common causes of renal disease in the general population, PLWH can also present with renal impairment secondary to ART or HIV-associated kidney disease [3–6]. Three out of the four ART regimens recommended by the Australian guidelines contain the nucleos(t)ide inhibitor tenofovir [7]. The older pro-drug of tenofovir—tenofovir disoproxil fumerate (TDF), has been known to cause a variety of adverse renal effects including proximal tubulopathy, decreased estimated glomerular filtration rate (eGFR) and proteinuria [3]. Consequently, the newer pro-drug—tenofovir alafenamide (TAF) has been developed and is replacing TDF, as it demonstrates lower rates of renal impairment—especially in people with lower starting eGFR [4, 5]. Two key examples of HIV-associated kidney disease are HIV-Associated Nephropathy (HIVAN) and HIV Immune Complex Kidney Disease (HIVICK). HIVAN is characterised histologically by collapsing Focal Segmental Glomerulosclerosis (FSGS) with tubular microcysts and interstitial inflammation [8]. Clinically, it presents with rapid renal impairment and marked proteinuria. HIVAN was first described in heavily immunocompromised, young African-American males in the 1980’s and has significantly decreased since the introduction of ART [9]. HIVICK can present as several common patterns of glomerular injury, including membranous or membranoproliferative glomerulonephritis and is characterised by immunoglobulin and/or immune-complex glomerular deposition [6].

Given the broad spectrum of kidney disease seen in PLWH, a renal biopsy is often pursued for accurate diagnosis and to guide clinical management. Ultrasound-guided renal biopsy is a relatively safe procedure that is commonly performed in people with ongoing renal impairment of uncertain aetiology. The risk of major bleeding with renal biopsy has been reported at 2.2%, with the incidence of death at 0.1% [10]. Evidence-based guidance on who and when to biopsy is lacking and therefore the choice to proceed to biopsy is often based on clinician experience. This study aims to describe biopsy-proven renal disease in PLWH at our institution and assess for any correlation between any demographic or laboratory characteristics and diagnoses, which may help guide when or when not to biopsy.

## Methods

A literature review of other renal biopsy studies in HIV patients was conducted using the National Library of Medicine PubMed service. The three key search phrases used for the search were “renal”, “biopsy” and “HIV”. Appropriate papers, published in English were included in Table 3.

A retrospective review of PLWH who underwent a renal biopsy between January 2010 and December 2020 at Royal Prince Alfred (RPA) Hospital, Sydney Australia was conducted. All PLWH who underwent a biopsy were included—unless they were younger than 18 years of age or had undergone previous kidney transplantation. There were no other strict exclusion criteria. Located in central Sydney, RPA is one of the busiest tertiary metropolitan teaching hospitals in New South Wales, seeing over seventy-five-thousand admission per year. It has all major medical and surgical specialties, including renal transplantation.

The electronic medical records of eligible PLWH who underwent biopsy were reviewed. Demographic information that was collected included: gender, age, country of origin, smoking status, illicit drug use and body mass index (BMI) (normal range 18.5 to 24.9 kg/m^2^). Smoking status and illicit drug use was determined from the documented history on eMR. BMI was automatically calculated (weight (kg) / height (m^2^) and reported on eMR. Relevant past medical history that was collected included: years on anti-retroviral therapy, current anti-retroviral therapy regimen, history of diabetes mellitus and history of hypertension. Years on anti-retroviral therapy was determined from the documented history on eMR. Anti-retroviral therapy regimen was listed in the medication section on eMR. Patients were noted to have a history of diabetes mellitus if the diagnosis was documented in the history, they were on oral or non-oral anti-hypoglycaemics or had a Haemoglobin A1c of greater than 6.5%. Patients were noted to have hypertension if it was documented in the history or if they were taking anti-hypertensives.

Laboratory data collected (with the laboratory normal range) included: creatinine (45–110 μmol/L), eGFR (> 90 mL/min/1.73m^2^), phosphate (0.75 to 1.50 mmol/L), potassium (3.5 to 5.2 mmol/L), corrected calcium (2.1 to 2.6 mmol/L), magnesium (0.7 to 1.1 mmol/L), HIV viral load (not detected), Haemoglobin A1c (HbA1c) (3.5 to 6.0%), absolute CD4 count (0.44 to 2.16 × 10^9^ cells / L), hepatitis B core antibody (negative), hepatitis C antibody (negative), syphilis antibodies and rapid plasma reagin (negative), urinary protein-to-creatinine ratio (PCR) (< 15 mg/mmol) and urinary albumin-to-creatinine ratio (ACR) (< 2.5 mg/mmol). All laboratory data used (including biochemistry, haematology, microbiology and serology) was taken from the day of the biopsy. If this was not available, the most recent laboratory test result was used. The eGFR was calculated using the CKD-EPI formula [11]. Categories of albuminuria were defined using the urinary Albumin-to-Creatinine Ratio (ACR): normal < 2.5 mg/mmol, microalbuminuria 2.5-25 mg/mol, macroalbuminuria 25-300 mg/mmol and nephrotic range > 300 mg/mmol.

The clinical features that lead to renal biopsy were evaluated using information provided on the pathology request form. Indicators for biopsy included decreased eGFR alone, proteinuria alone, decreased eGFR plus proteinuria, decreased eGFR plus proteinuria plus haematuria and proteinuria plus haematuria.

Each renal biopsy was analysed and reported by a consultant pathologist at the Central Sydney Local Health District Laboratory Service (attached to RPA). Light microscopy, immunofluorescence and electron microscopy were performed on all cohort samples. If there was more than one histological diagnosis on the biopsy, both were included in the analysis.

Statistical analysis was performed using the SPSS package (Version 26, IBM, New York, USA) and figures used were created using Prism (Version 9, GraphPad, California, USA). The point estimates presented in figures are either mean (standard deviation) or number of cases (percentage of total) unless otherwise specified. An association between two categorical variables was assessed using a chi-square test and between a categorical and a continuous variable using Kendall’s coefficient of rank correlation. For all he analyses, a p value of < 0.05 was considered statistically significant.

This study was conducted in accordance with ethical standards presented in the declaration of Helsinki. Ethics approval was gained from the Human Research Ethics Committee of the Sydney Local Health district.

## Results

Between January 2010 and December 2020, there were 481 renal biopsies performed at our institution, 19 of which were performed on PLWH and were eligible for inclusion in the study. The number of biopsies per year ranged from zero to five during the 10-year period.

The demographic and clinical characteristics of our cohort are outlined in Table 1. The majority of PLWH were Australian born (53%), male (84%) and had a mean age of 48 years (SD 13). Almost half of the cohort (47%) were current smokers, most (68%) did not use any illicit substances and the mean BMI for the cohort was 26 kg/m^2^ (SD 6). Almost all (95%) people were on ART therapy, with a mean treatment duration of 9 years (SD 8). A TAF-based regimen was used by 47% of the cohort, whereas only 16% were on a TDF-containing regimen. Hypertension was present in three quarters (74%) of the people and one fifth (21%) of the cohort had diabetes mellitus.

**Table 1:** Demographic and clinical characteristics of People Living With HIV at the time of biopsy. (N) denotes the number of people with available data. Body mass index (BMI), Highly Active Anti-Retroviral therapy (ART), Tenofovir Disoproxil Fumerate (TDF), Tenofovir Alafenamide (TAF)

**Characteristic (N)**		**Mean (SD) or n (%)**
Gender (19)	Male	16 (84%)
	Female	3 (16%)
Age (19)		48 years (SD 13)
Country of Origin (19)	Australia	10 (53%)
	India	2 (11%)
	China	2 (11%)
	Thailand	1 (5%)
	Hungary	1 (5%)
	Philippines	1 (5%)
	Samoa	1 (5%)
	America	1 (5%)
Smoking status (15)	Current smoker	7 (47%)
	Ex-smoker	2 (13%)
	Never smoked	6 (40%)
Illicit drug use (12)	Ecstasy	1 (8%)
	Amphetamines	1 (8%)
	Ecstasy and amphetamines	1 (8%)
	Heroin	1 (8%)
	None	8 (68%)
BMI (15)		26 kg/m^2^ (SD 6)
Years on ART (15)		9 years (SD 8)
Tenofovir-based ART (19)	TDF	3 (16%)
	TAF	9 (47%)
	non-tenofovir ART	6 (32%)
	nil ART	1 (5%)
Co-morbidities (19)	Diabetes Mellitus	4 (21%)
	Hypertension	15 (74%)
Clinical Features (19)		
	Decreased eGFR + proteinuria + haematuria	6 (32%)
	Decreased eGFR + proteinuria	5 (26%)
	Proteinuria + haematuria	3 (16%)
	Decreased eGFR alone	3 (16%)
	Proteinuria alone	2 (10%)

The most common clinical indicator for biopsy was the combination of decreased eGFR, proteinuria and haematuria (32%) (see Table 1). Decreased eGFR and proteinuria alone were the second most common clinical features (26%). No people were biopsied purely for the workup of hypertension. The indication listed for biopsy in six cases was specifically differentiating between ART/HIV-associated kidney disease and diabetic or hypertensive nephropathy.

The laboratory results for the cohort at the time of biopsy are detailed in Table 2. The mean serum creatinine was 132 µmol/L (SD 55) and the mean eGFR was 61 ml/min/1.73m^2^ (SD 24). The mean potassium and magnesium were within the normal range. There were no instances of hypocalcaemia or hypophosphataemia. Only 17% of the cohort had a normal ACR. Most people had a macroalbuminuria (61%) and over a fifth (22%) had nephrotic range proteinuria. The mean absolute CD4 count was 0.58 × 10^9^ cells/L (SD 0.31) and over half (58%) of the cohort had an undetectable viral load. Prior Hepatitis B exposure was common (37%) and 16% were positive for hepatitis C antibodies. The mean HbA1c was elevated at 7.4% (SD 3.4). Positive syphilis antibody screen and rapid plasma regain was noted in one patient, but data was only available for 9 people.

**Table 2:** Laboratory values of People Living With HIV at time of biopsy. (N) denotes the number of people with available data. The Laboratory reference range is as published by Sydney Local Health District and utilised at the Royal Prince Alfred Hospital.—Estimated glomerular filtration rate (eGFR), haemoglobin A1C (HbA1C), Protein-to-Creatinine Ratio (PCR), Albumin-to-Creatinine Ratio (ACR)

Laboratory Values (n)	Mean (SD) or n (%)	Laboratory Reference Range
Creatinine (19)	132 (55)	45–110 μmol/L
eGFR (19)	61 (24)	> 90 mL/min/1.73m^2^
Phosphate (17)	1.15 (0.18)	0.75 to 1.50 mmol/L
Potassium (19)	4.5 (0.4)	3.5 to 5.2 mmol/L
Corrected calcium (17)	2.4 (0.1)	2.1 to 2.6 mmol/L
Magnesium (18)	0.8 (0.1)	0.7 to 1.1 mmol/L
Viral load (19)		
not detected	11 (58%)	-
< 50 copies	5 (26%)	
50–100 copies	3 (16%)	
HbA1C (4)	7.4 (3.4)	3.5 to 6.0%
Absolute CD4 count (19)	0.58 (0.31)	0.44 to 2.16 × 10^9^ cells / L
Hepatitis B core antibody positive (18)	5 (28%)	-
Hepatitis C antibody positive (19)	3 (16%)	-
Syphilis antibody positive (9)	1 (11%)	-
PCR (14)	226 (327)	< 15 mg/mmol
ACR (18)		
Normal	3 (17%)	< 2.5 mg/mmol
Microalbuminuria	4 (22%)	
Macroalbuminuria	11 (61%)	
Nephrotic	4 (22%)	

**Fig. 1:**
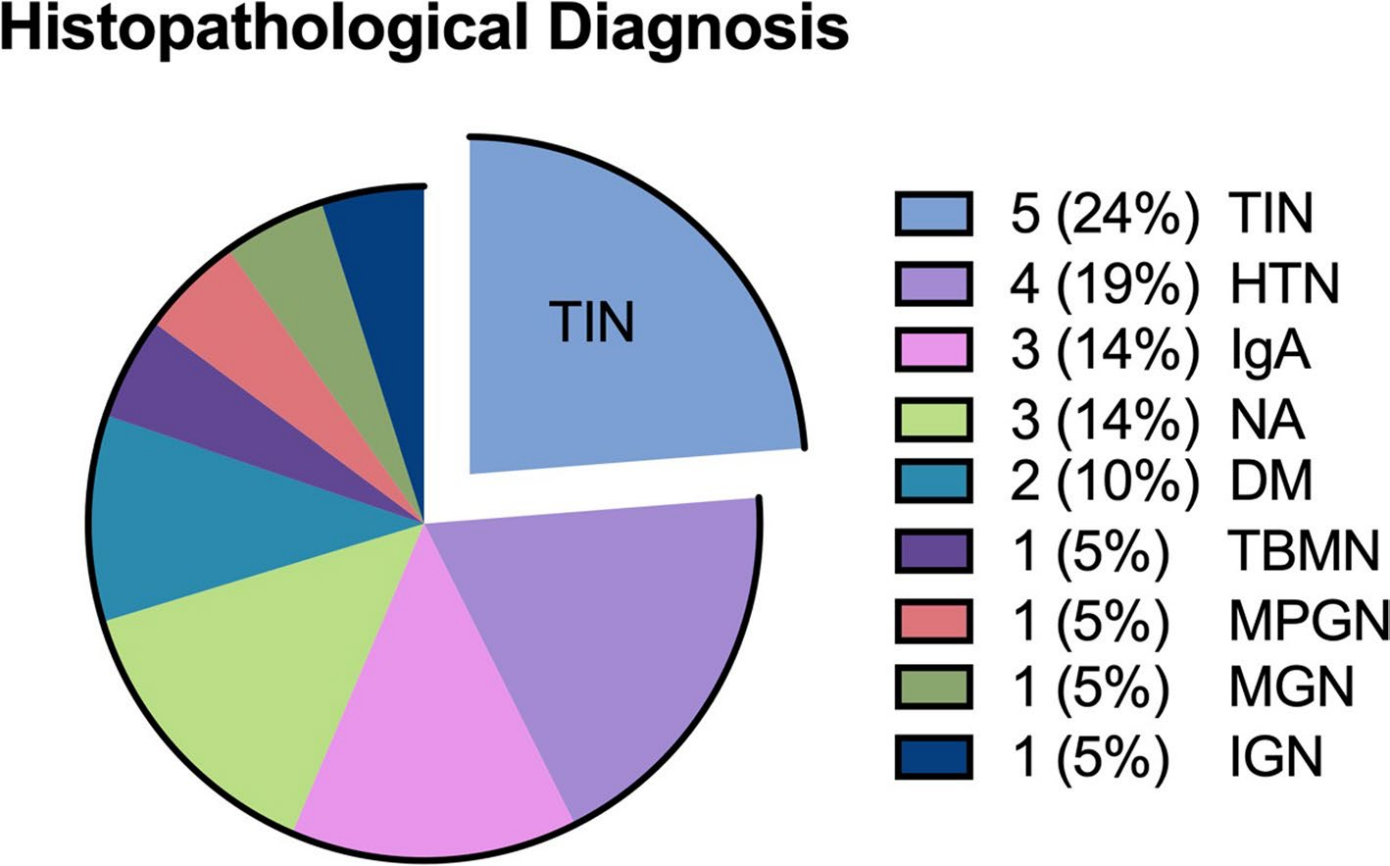
Renal biopsy diagnoses. Numbers represent percentage of the twenty-one diagnoses made on renal biopsies analysed in study.—Tubulointerstital Nephritis (TIN), Hypertension (HTN), Immunoglobulin A nephropathy (IgA), No abnormality found (NA), Diabetic Nephropathy (DM), Thin Basement Membrane Nephropathy (TBMN), Membranoproliferative glomerulonephritis (MPGN), Membranous glomerulonephritis (MGN), Ischaemic glomerulonephropathy (IGN)

There was a total of 21 histological diagnoses made on the 19 renal biopsies. The most common diagnosis amongst our cohort was tubulointerstitial nephritis (TIN) – which was seen in five people (24%) (Fig. 1). This was followed by hypertensive glomerulosclerosis in 4 people (19%). IgA nephropathy and no abnormality were seen in 3 people each (14%). Diabetic nephropathy was present in 2 people (10%) and thin-membrane nephropathy, membranoproliferative glomerulonephritis, membranous glomerulonephritis and ischaemic glomerulosclerosis were only seen in one person each (5%). No people had evidence of collapsing FSGS to suggest HIVAN on renal biopsy. One biopsy had both interstitial nephritis and diabetic nephropathy and one biopsy had both diabetic nephropathy and hypertensive glomerulosclerosis.

There was no association between any clinically relevant demographic or laboratory characteristic and histological diagnosis: age (p 0.49), gender (p 0.17), country of origin (p 0.46), years on ART therapy (p 0.53), type of tenofovir (p 0.574), BMI (p 0.36), diabetes mellitus (p 0.179), hypertension (p 0.54), creatinine (p 0.47), eGFR (p 0.74), HbA1C (p 0.72), viral load (p 0.45), absolute CD4 count (p 0.33), hepatitis B core antibody positive (p 0.39), hepatitis C antibody positive (p 0.255), syphilis antibody positive (p 0.41) and albuminuria (p 0.34).

## Discussion

This study represents the first description of biopsy-proven kidney disease in the HIV-infected population of Australia. Our results support the use of renal biopsy in PLWH with ongoing renal impairment for accurate diagnosis and to guide further management.

The majority of previously published international biopsy studies have reported on similarly small cohort numbers to our study – highlighting the generally low incidence of renal biopsy in this population (Table 3). Two exceptions are the large cohorts reported on in the United States and South Africa [12, 13]. However, their findings may not be generalisable to the Australian cohort due to the large population of African individuals, who are at a higher risk of HIV-associated kidney disease, especially HIVAN [9].

**Table 3:** Summary of previous renal biopsy studies in People Living With HIV. “not listed” means that the study did not report on the specific variable, “nil” means there was no cases.—Diabetes Mellitus (DM), Hypertension (HTN), Hepatitis B core positive (Hep B), Hepatitis C antibody positive (Hep C), Tubulointerstitial nephritis (TIN), membranoproliferative glomerulonephritis (MPGN), Human immunodeficiency virus associated Nephritis (HIVAN), diabetic nephropathy (DN), Haemolytic-ureamic syndrome (HUS), Immune-complex disease (ICD), Acute Interstitial Nephritis (AIN) and Immunoglobulin A nephropathy (IgA)

**Paper**	**Year**	**Country**	**Number of patients**	**Mean Age**	**Serum Creatinine**	**DM**	**HTN**	**Hep B**	**Hep C**	**Most Common Diagnosis**	**TIN**	**HIVAN**	**ICD**	**DN**	**HTN**
This study	2021	Australia	19	47.9	1.49 mg/dL	4 (21.1%)	14 (73.7%)	7 (36.8%)	3 (15.8%)	TIN 5 (24%)	4 (21%)	nil	IgA 3 (14%)	2 (8%)	4 (17%)
Praditopornsilpa [14]	1999	Thailand	26	31	2.36 mg/dL	excluded	nil	exlcuded	exlcuded	MPGN 17 (65%)	17 (65%)	nil	IgA 2 (7.6%)	nil	nil
Berliner [12]	2004	United states	152	42.6	4.1 mg/dL	26 (17.1%)	92 (62%)	12 (8.4%)	86 (59.3%)	HIVAN 53 (34.9%)	12 (7.9%)	53 (34.9%)	IgA 3 (2%)	8 (5.3%)	5 (3.3%)
Singh [15]	2019	India	10	34.1	1.84 mg/dL	not listed	not listed	not listed	not listed	MPGN 5 (50%)	2 (20%)	2 (20%)	nil	1 (10%)	nil
Hara [16]	2018	Japan	10	54	1.56 mg/dL	4 (40%)	5 (50%)	2 (20%)	2 (20%)	DN 3 (33%)	2 (20%)	nil	nil	3 (33%)	nil
Vermeulen [13]	2019	South Africa	1848	33.5	3.57 mg/dL	not listed	not listed	83 (4.5%)	28 (1.5%)	HIVAN 604 (32.7%)	70 (2.8%)	604 (32.7%)	ICD 218 (11.8%)	81 (4.4%)	55 (3%)
Williams [17]	1998	England	17	37.8	not listed	not listed	not listed	4 (23%)	not listed	HIVAN 7 (41%)	1 (5.8%)	7 (41%)	IgA 1 (5.8%)	nil	nil
Peraldi [18]	1999	France	60	35.1	5.43 mg/dL	not listed	11 (18.3%)	1 (1.6%)	1 (1.6%)	HUS 32 (35%)	18 ( 19.5%)	14 (15.2%)	ICD 2 (2.17%)	nil	nil
Nebuloni [19]	2009	Italy	73	not listed	not listed	not listed	not listed	not listed	not listed	ICD 40 (54.7%)	nil	9 (12.3%)	ICD 40 (54.7%)	4 (5.4%)	nil
Schmid [20]	2007	Switzerland	30	35	not listed	not listed	not listed	not listed	2 (6.6%)	ICD 13 (43%)	6 (20%)	2 (6.6%)	ICD 13 (43%)	1 (3.3%)	1 (3.3%)

Previous hepatitis B exposure was noted in seven people within our cohort (37%) and three (16%) had positive hepatitis C antibodies. Interestingly, none of the cohort with prior hepatitis B or hepatitis C exposure, had the typical membranous or membranoproliferative glomerulonephritis associated with these infections. Unfortunately, hepatitis viral load and past/current treatment data was unavailable to assess if this could be due to historical exposure versus current infection. Interestingly, one patient was noted to have positive syphilis serology, however their biopsy was consistent with hypertensive and diabetic change and so their infection was unlikely to have contributed.

Our cohort had an older mean age (48 years) than almost all other international studies. This may explain the higher rate of co-morbid diabetes and hypertension in our study, compared with the younger populations such as in Thailand and France [14, 18]. Despite the high prevalence of comorbid conditions, our cohort had less renal impairment (in terms of eGFR) at time of biopsy, compared with other international studies. This may imply that either our cohort were biopsied earlier in their disease course or that the renal pathology affecting our cohort, produced less of a profound azotaemia compared with the other studies. Notably, the most common diagnosis in studies with the lowest eGFR was HIVAN, known to cause severe renal azotaemia [8, 9].

The most common histological diagnosis observed in our study was tubulointerstitial nephritis (TIN)—characterised by oedema, interstitial mononuclear cellular infiltrate and tubulitis on histology [21]. Most cases of TIN are non-infectious and are associated with drug therapy; antibiotics, proton pump inhibitors and nonsteroidal anti-inflammatories are most frequently implicated [22]. Unfortunately, we did not have data on our cohort’s non-ART medications to test for these associations. Although less frequently observed, infections with Cytomegalovirus Virus, Epstein Barr Virus and Mycobacterium Tuberculosis can also contribute to the development of TIN [22]. Low rates of active infection with these agents in the Australian HIV population (post-introduction of ART) and the exclusion of people post transplantation from our study, make these unlikely to be contributory [23, 24]. Interestingly, twenty percent of biopsies in the Zurich cohort showed TIN, especially in PLWH on the combination of tenofovir and atazanavir [20]. Our study found no association between ART regimen and diagnoses and those with TIN in our cohort were either on tenofovir or atazanavir, but not both. The mainstay of management for TIN is withdrawal of any suspected drug(s), treatment of any systemic infections and use of corticosteroids [25]. Early administration of corticosteroids has been associated with at least partial recovery of renal function in over 80% of TIN cases, with benefits diminishing if treatment is delayed [25]. Thus, people with TIN, need to be identified early and have corticosteroid therapy initiated to achieve the best renal outcomes.

The second most common histological diagnosis in our study was hypertensive glomerulosclerosis. This is unsurprising, given that 74% of our cohort suffered from hypertension. Compared to other international studies, our study had the highest rate of comorbid hypertension (Table 3). This could be secondary to the older mean age of our population, compared to most other studies. Interestingly, papers from the United States and Japan both reported rates of comorbid hypertension above 50% and yet hypertensive nephrosclerosis was seen in less than 4% of their cohort [12, 16]. This may suggest that blood pressure control in the other cohorts was tighter than in our study, although without longitudinal blood pressure data this is hard to assess.

Although there were 4 people (8%) with comorbid diabetes in our study, only two demonstrated evidence of diabetic nephropathy on histology. There was also no correlation between comorbid diabetes or HbA1c with a histological diagnosis of diabetic nephropathy. This was likely secondary to the small sample size or may be due to historical kidney damage done before their diabetes was under control and their HbA1c decreased. Our results contrasted with the Japanese cohort, where all people with diabetes demonstrated diabetic nephropathy on biopsy [16]. Acknowledging the small cohort numbers, these differences may be explained by the older cohort and Asian ethnicity, which are associated with a higher rate of diabetic complications [26].

Compared with other studies, our cohort had the highest proportion of IgA deposition on immunofluorescence (14%), however this was consistent with the rate of IgA nephropathy in the general population [27]. Other predominately Caucasian and Asian countries, such as Thailand, United states and England also had similar rates [12, 14, 17]. There were no instances of IgA nephropathy seen in the African or Indian population, which is concordant with what has been reported before [28]. The key management strategy for IgA nephropathy is blood pressure control with renin-angiotensin aldosterone blockade [29].

No people in our cohort demonstrated histological features of HIVAN on biopsy. This was in contrast to other international studies—especially in United states and South Africa where HIVAN was the most common finding [12, 13]. This is likely explained by the low prevalence of African ethnicity in the Australian population [30]. The results from both our study and other international cohorts, suggest that the rate of HIVAN is not explained by absolute CD4 count alone (Table 3). Analogously to the association of markers of diabetes with diabetic nephropathy, the absolute CD4 count at the time of biopsy may not reflect counts during the first years of infection, when renal injury may have occurred. Studies from England and France had equally low CD4 counts and yet the rate of HIVAN was more than double in England [17, 18]. Similarly, studies from Thailand and South African had equivalent CD4 counts, yet HIVAN was seen in over 30% of the South African population, while no cases were seen in Thailand [13, 14].

Although there was one instance of membranous and membranoproliferative glomerulonephritis on biopsy, none of these demonstrated deposition of immunoglobulins or complexes to suggest HIVICK. However, the development of HIVICK is most associated with African ethnicity so this is unsurprising in the Australian setting [30]. Further characterisation of the natural history and risk factors for development of HIVICK is required.

Given no demographic or laboratory variables were found to have a significantl association with any histological diagnosis, it is unlikely any individual clinical characteristic can help steer the clinician to the underlying aetiology of the renal impairment without biopsy.

There were several limitations to our study. Firstly, the limited number of renal biopsies performed in the HIV population at our institution limited the generalisability of the results. It also impacted our ability to find correlation between demographic and laboratory characteristics with histological diagnoses. However, given our study had a larger cohort size than several other internationally published papers, it still adds to the growing literature in this unique population. Secondly, non-ART medications and other comorbidities (beside hypertension and diabetes) were not able to be collected and analysed. This could have allowed further analysis of risk factors for the development of TIN in our cohort. Lastly, there was the possibility of selection bias as it was up to the discretion of the individual renal physician which people underwent biopsy and there were no strict inclusion criteria. This could have resulted in people with more severe renal impairment being biopsied, revealing diagnoses that were different from those with less severe renal impairment.

## Conclusions

This study found that tubulointerstitial nephritis, hypertensive glomerulosclerosis and IgA nephropathy were the most common histological findings on renal biopsy within the HIV population at our institution. If there are features of TIN on biopsy, there is good evidence to support the use of corticosteroids to achieve renal recovery. However, the finding of hypertensive glomerulosclerosis or IgA nephropathy should prompt the clinician to concentrate on blood pressure control to prevent progression. There was no correlation between demographic or laboratory characteristics with histological diagnosis. Thus, renal biopsy remains crucial in the Australian HIV population to ensure the correct diagnosis and determine the best clinical management.

## Data Availability

The datasets generated and/or analysed during the current study are not publicly available due to local health policy to not share any individual research results but are available from the corresponding author on reasonable request.

## Abbreviations

ACR: Albumin-to-Creatinine Ratio
ART: Anti-Retroviral Therapy
BMI: Body Mass Index
CD4: Cluster of Differentiation 4
eGFR: Estimated Glomerular Filtration rate
FSGS: Focal Segmental Glomerulosclerosis
HbA1c: Haemoglobin A1c
HIV: Human Immunodeficiency Virus
HIVAN: Human Immunodeficiency Virus Associated Nephropathy
HIVAN: Human Immunodeficiency Virus Immune Complex Kidney Disease
IgA: Immunoglobulin A
PLWH: People Living With HIV
PCR: Protein-to-Creatinine Ratio
SD: Standard Deviation
TAF: Tenofovir Alafenamide Fumerate
TDF: Tenofovir Disoproxil Fumerate
TIN: Tubulointerstitial Nephritis

## References

[1] The Kirby Institute for infection and immunity in society, UNSW Sydney, Sydney, NSW (2020). National update on HIV, viral hepatitis and sexually transmissible infections in Australia: 2009–2018.

[2] Cheung J, Puhr R, Petoumenos K, Cooper DA, Woolley I, Gunathilake M (2018). Chronic kidney disease in Australian Human Immunodeficiency Virus-infected patients: Analysis of the Australian HIV Observational Database. Nephrology (Carlton).

[3] Scherzer R, Estrella M, Li Y, Choi AI, Deeks SG, Grunfeld C (2012). Association of tenofovir exposure with kidney disease risk in HIV infection. AIDS.

[4] Gallant JE, Daar ES, Raffi F, Brinson C, Ruane P, DeJesus E (2016). Efficacy and safety of tenofovir alafenamide versus tenofovir disoproxil fumarate given as fixed-dose combinations containing emtricitabine as backbones for treatment of HIV-1 infection in virologically suppressed adults: a randomised, double-blind, active-controlled phase 3 trial. Lancet HIV.

[5] Turner D, Drak D, O’Connor CC, Templeton DJ, Gracey DM (2019). Renal function change after switching tenofovir disoproxil fumarate for tenofovir alafenamide in the HIV-positive patients of a metropolitan sexual health service. AIDS Res Ther.

[6] Booth JW, Hamzah L, Jose S, Horsfield C, O'Donnell P, McAdoo S, Kumar EA, Turner-Stokes T, Khatib N, Das P, Naftalin C, Mackie N, Kingdon E, Williams D, Hendry BM, Sabin C, Jones R, Levy J, Hilton R, Connolly J, Post FA; HIV/CKD Study and the UK CHIC Study. Clinical characteristics and outcomes of HIV-associated immune complex kidney disease. Nephrol Dial Transplant. 2016;31(12):2099–107. 10.1093/ndt/gfv436. Epub 2016 Jan 18. PMID: 26786550. 10.1093/ndt/gfv436 26786550

[7] McMahon J, Grulich A, Whittaker B, Burnett C, Collins D, Nolan D et al. Antiretroviral Guidelines: US DHSS Guidelines with Australian Commentary 2019 [Internet]. 2019. Available from: https://arv.ashm.org.au/.

[8] Laurinavicius A, Hurwitz S, Rennke HG (1999). Collapsing glomerulopathy in HIV and non-HIV patients: a clinicopathological and follow-up study. Kidney Int.

[9] Abbott KC, Hypolite I, Welch PG, Agodoa LY (2001). Human immunodeficiency virus/acquired immunodeficiency syndrome-associated nephropathy at end-stage renal disease in the United States: patient characteristics and survival in the pre highly active antiretroviral therapy era. J Nephrol.

[10] Lees J, McQuarrie E, Mordi N, Geddes C, Fox J, Mackinnon B (2017). Risk factors for bleeding complications after nephrologist-performed native renal biopsy. Clin Kidney J.

[11] Levey AS, Stevens LA, Schmid CH, Zhang YL, Castro AF, Feldman HI (2009). A new equation to estimate glomerular filtration rate. Ann Intern Med.

[12] Berliner AR, Fine DM, Lucas GM, Rahman MH, Racusen LC, Scheel PJ (2008). Observations on a cohort of HIV-infected patients undergoing native renal biopsy. Am J Nephrol.

[13] Vermeulen A, Menezes CN, Mashabane M, Butler OK, Mosiane P, Goetsch S (2019). Patterns of renal disease: A 30-year renal biopsy study at Chris Hani Baragwanath Academic Hospital, Soweto, Johannesburg. South Africa S Afr Med J.

[14] Praditpornsilpa K, Napathorn S, Yenrudi S, Wankrairot P, Tungsaga K, Sitprija V (1999). Renal pathology and HIV infection in Thailand. Am J Kidney Dis.

[15] Verma B, Singh A (2019). Histological spectrum of renal disease in HIV/AIDS patients with significant proteinuria: An Indian perspective. J Family Med Prim Care.

[16] Hara M, Momoki K, Ubukata M, Ohta A, Tonooka A, Ando M (2018). The renal pathological findings in Japanese HIV-infected individuals with CKD: a clinical case series from a single center. Clin Exp Nephrol.

[17] Williams DI, Williams DJ, Williams IG, Unwin RJ, Griffiths MH, Miller RF (1998). Presentation, pathology, and outcome of HIV associated renal disease in a specialist centre for HIV/AIDS. Sex Transm Infect.

[18] Peraldi MN, Maslo C, Akposso K, Mougenot B, Rondeau E, Sraer JD (1999). Acute renal failure in the course of HIV infection: a single-institution retrospective study of ninety-two patients and sixty renal biopsies. Nephrol Dial Transplant.

[19] Nebuloni M, di BarbianoBelgiojoso G, Genderini A, Tosoni A, Riani NL (2009). Glomerular lesions in HIV-positive patients: a 20-year biopsy experience from Northern Italy. Clin Nephrol.

[20] Schmid S, Opravil M, Moddel M, Huber M, Pfammatter R, Keusch G, Ambuhl P, Wuthrich RP, Moch H, Varga Z (2007). Acute interstitial nephritis of HIV-positive patients under atazanavir and tenofovir therapy in a retrospective analysis of kidney biopsies. Virchows Arch.

[21] Fogo AB, Lusco MA, Najafian B, Alpers CE (2016). AJKD atlas of renal pathology: Acute interstitial nephritis. Am J Kidney Dis.

[22] Muriithi AK, Leung N, Valeri AM, Cornell LD, Sethi S, Fidler ME (2014). Biopsy-proven acute interstitial nephritis, 1993–2011: a case series. Am J Kidney Dis.

[23] Seale H, Macintyre CR, Dwyer DE, Wang H (2007). The changing epidemiology of severe cytomegalovirus disease in Australia. Hum Vaccin.

[24] World Health Organisation. Latent tuberculosis infection: updated and consolidated guidelines for programmatic management [Internet]. 2018. Available from: https://apps.who.int/iris/handle/10665/260233 30277688

[25] Fernandez-Juarez G, Perez JV, Caravaca-Fontán F, Quintana L, Shabaka A, Rodriguez E (2018). Duration of treatment with corticosteroids and recovery of kidney function in acute interstitial nephritis. Clin J Am Soc Nephrol.

[26] Karter AJ, Ferrara A, Liu JY, Moffet HH, Ackerson LM, Selby JV (2002). Ethnic disparities in diabetic complications in an insured population. JAMA.

[27] Waldherr R, Rambausek M, Duncker WD, Ritz E (1989). Frequency of mesangial IgA deposits in a non-selected autopsy series. Nephrol Dial Transplant.

[28] Crowley-Nowick PA, Julian BA, Wyatt RJ, Galla JH, Wall BM, Warnock DG (1991). IgA nephropathy in blacks: studies of IgA2 allotypes and clinical course. Kidney Int.

[29] Kidney Disease: Improving Global Outcomes (KDIGO) Glomerulonephritis Work Group (2012). KDIGO Clinical Practice Guideline for Glomerulonephritis. Kidney Inter Suppl.

[30] Foy MC, Estrella MM, Lucas GM, Tahir F, Fine DM, Moore RD (2013). Comparison of risk factors and outcomes in HIV immune complex kidney disease and HIV-associated nephropathy. Clin J Am Soc Nephrol.

